# Kinematics and kinetics comparison of ultra-congruent versus medial-pivot designs for total knee arthroplasty by multibody analysis

**DOI:** 10.1038/s41598-022-06909-x

**Published:** 2022-02-23

**Authors:** Giovanni Putame, Mara Terzini, Fabrizio Rivera, Maeruan Kebbach, Rainer Bader, Cristina Bignardi

**Affiliations:** 1 Department of Mechanical and Aerospace Engineering, Politecnico di Torino, Turin, Italy; 2 PolitoBIOMed Lab, Politecnico di Torino, Turin, Italy; 3 Orthopaedic Surgery Department, SS Annunziata Savigliano Hospital, Azienda Sanitaria Locale CN1, Savigliano, Italy; 4 Department of Orthopaedics, Rostock University Medical Center, Rostock, Germany

**Keywords:** Biomedical engineering, Computational science

## Abstract

Nowadays, several configurations of total knee arthroplasty (TKA) implants are commercially available whose designs resulted from clinical and biomechanical considerations. Previous research activities led to the development of the so-called medial-pivot (MP) design. However, the actual benefits of the MP, with respect to other prosthesis designs, are still not well understood. The present work compares the impact of two insert geometries, namely the ultra-congruent (UC) and medial-pivot (MP), on the biomechanical behaviour of a bicondylar total knee endoprosthesis. For this purpose, a multibody model of a lower limb was created alternatively integrating the two implants having the insert geometry discretized. Joint dynamics and contact pressure distributions were evaluated by simulating a squat motion. Results showed a similar tibial internal rotation range of about 3.5°, but an early rotation occurs for the MP design. Furthermore, the discretization of the insert geometry allowed to efficiently derive the contact pressure distributions, directly within the multibody simulation framework, reporting peak pressure values of 33 MPa and 20 MPa for the UC and MP, respectively. Clinically, the presented findings confirm the possibility, through a MP design, to achieve a more natural joint kinematics, consequently improving the post-operative patient satisfaction and potentially reducing the occurrence of phenomena leading to the insert loosening.

## Introduction

Since the early 1970s, increasingly more sophisticated designs of total knee endoprostheses have been developed thanks to the collaboration between surgeons and bioengineers^1^. Among the several features that have to be taken into account during the implant design process, there are, for instance, the range of motion, the modularity of the implant components, the type of insert fixation, and last but not least, the geometrical congruence between articulating surfaces^2^. In particular, this last aspect represents a critical factor since tibiofemoral implant conformity must guarantee joint stability and, at the same time, minimize contact force concentrations to prevent excessive wear at the contact interfaces. Indeed, the insert design assumes a major role in defining the artificial knee kinematics, which is related to wear performance and, consequently, to the longevity of the total knee replacement^3^. Although new materials and design concepts have been introduced, complications related to joint instability and insert wear are still common causes of revision surgery^4^. Focusing on implant kinematics, since the late 1990s, the trend in implant design aimed to mimic the physiological medial-pivoting movement of the knee, which is combined with a rollback of the lateral femoral condyle. Even though literature reports good surgical and kinematical outcomes using the MP design^5–7^, it is unclear whether this design is significantly related to clinical and biomechanical improvements with respect to traditional designs. Therefore, further investigations are needed to achieve a better understanding of the different implants’ performance.

In this scenario, the possibility to predict in vivo kinematics and loading conditions of the artificial knee is essential to assess implant performance and improve the implant design as well as the surgical outcomes in TKA. Computational modelling can provide useful information about joint contact forces and kinematics to identify detrimental loading conditions that could lead to failure of the knee implant^3,8,9^. Specifically, musculoskeletal multibody models have proven to be able to reproduce realistic dynamics of the knee joint, besides being particularly suitable for experimental validations^10–15^.

In this study, the biomechanical behaviour of two different insert designs, namely, the UC and the MP was compared within a multibody model of a lower limb. The kinematics and kinetics of the artificial knee joint were evaluated by simulating a weight-bearing deep squat motion. In particular, the contact force and pressure distributions on the inserts were investigated by discretizing their geometry. The hypothesis underlying the study is that the MP design leads to higher internal/external tibial rotations and lower constraining forces compared to the UC design. From a clinical standpoint, a confirmation of this hypothesis, would correspond to a physiological-like kinematics and kinetics, and, consequently, to an improvement of the post-operative patient satisfaction as well as a potential lower risk of implant failure due to loosening.

## Methods

### Geometries

The multibody model of a right lower limb was created in the multibody dynamic analysis program ADAMS (v. 2017, MSC Software Corporation, Santa Ana, CA) assembling standardized (medium size) bone geometries (Sawbones^®^ Europe AB, Malmoe, Sweden) equipped with a bicondylar total knee implant (K-MOD, Gruppo Bioimpianti, Peschiera Borromeo, Milan, Italy) including two alternative insert designs, that are, the UC and MP. Specifically, a mechanical alignment approach was used for the implant positioning with a posterior tibial slope equal to 3°.

Bone geometries were transformed to fit on patient-specific geometries available in the literature^16,17^ corresponding to a male subject, who received a right knee prosthesis. More precisely, the center of the femoral head, the ankle midpoint, and the surgical cutting planes at the knee joint, were taken as references during the fitting process of femoral and tibial bones. Moreover, the patella was represented by an ellipsoid, while the fibula was transformed in accordance with the tibia. In addition, pelvis and foot bones were scaled and included as well.

Furthermore, the implant geometries were scaled based on the manufacturer’s sizes (i.e., size 5) to match the patient-specific bones. In details, the dual radius femoral component and both considered inserts were isotropically scaled up by a scale factor equal to 1.21, whereas the tibial tray was scaled up by a scale factor of 1.14, except for the normal direction to the component plateau which was not modified.

### Ligaments

**Figure 1:**
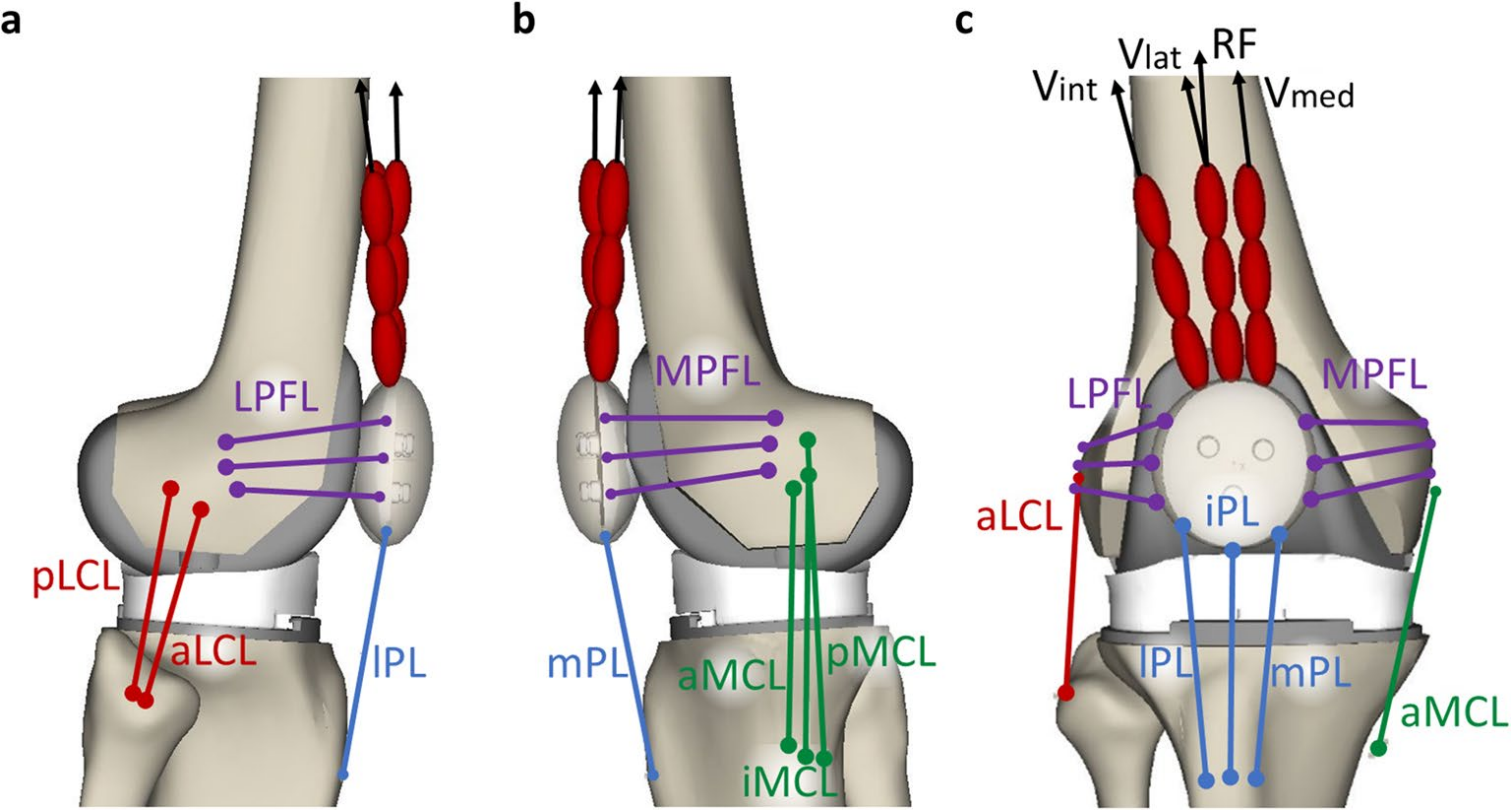
Ligament bundles and muscles included into the multibody model. (**a**) Lateral view; (**b**) medial view; (**c**) frontal view. Red ellipsoids represent tendons of the M. quadriceps femoris and black upward arrows indicate the initial action lines of the Vastus medialis (Vmed), Vastus lateralis (Vlat), Vastus intermedius (Vint), and Rectus femoris (RF).

The following ligament structures were included in the model: medial (MCL) and lateral (LCL) collateral ligaments, medial (MPFL) and lateral (LPFL) patellofemoral ligaments, and patellar ligament (PL). The anterior and posterior cruciate ligaments were not included because the first is sacrificed during the surgical procedure, whereas the latter can be excluded since both insert geometries were designed to permit this choice^7^. Each ligament was furtherly split into different bundles, in detail: MCL anterior (aMCL), intermediate (iMCL) and posterior (pMCL) bundle; LCL anterior (aLCL) and posterior (pLCL) bundle; LPFL proximal (pLPFL), middle (mLPFL) and distal (dLPFL) bundle; MPFL proximal (pMPFL), middle (mMPFL) and distal (dMPFL) bundle; PL medial (mPL), intermediate (iPL) and lateral (lPL). This division allows considering the ligament structure in bundles with their different constraining contribution over the joint movement. Origin and insertion points (Fig. 1) were determined from anatomical references^18^.

Each ligament bundle was modelled as a single tension-only spring element connecting the origin and insertion points^19^. In detail, the force-strain relationship of each spring is described by a nonlinear piecewise function (Eq. 1), where *ε* is the ligament strain, *ε*_*L*_ is a reference value of strain assumed to be 0.03 and *k* is the stiffness parameter, expressed as force per unit strain, of each different ligament bundle. The ligament strain *ε* is defined by Eq. (2), where *l* is the actual ligament length and *l*_*0*_ is the zero-load length, also called slack length, that is, the maximum linear distance between the ligament attachment points above which the ligament gets taut.

1 $$f = \left\{ {\begin{array}{*{20}l} { - k\left( {\varepsilon - \varepsilon_{L} } \right),} & {\quad \varepsilon > 2\varepsilon_{L} } \\ { - 0.25 k\frac{{\varepsilon^{2} }}{{\varepsilon_{L} }},} & {\quad 0 \le \varepsilon \le 2\varepsilon_{L} } \\ {0,} & {\quad \varepsilon < 0} \\ \end{array} } \right.$$

2 $$\varepsilon = \frac{{\left( {l - l_{0} } \right)}}{{l_{0} }}$$

The considered ligaments bundles with the related stiffness parameters *k* (Table 1) were retrieved from literature studies^10,20–24^.

**Table 1:** Stiffness parameters (expressed as force per unit strain) for each ligament bundle.

Ligament bundle	*k* (N)	
aMCL	2500	^10^
iMCL	3000	^10^
pMCL	2500	^10^
aLCL	2000	^10,20^
pLCL	2000	^10,20^
lPL	58,000	^21^
iPL	58,000	^21^
mPL	58,000	^21^
pMPFL	141	^23,24^
mMPFL	141	^23,24^
dMPFL	141	^23,24^
pLPFL	141	^23,24^
mLPFL	141	^23,24^
dLPFL	141	^23,24^

The zero-load length of each ligament bundle was tuned by iteratively performing the simulation of a passive knee flexion. For this purpose, the femoral and tibial segments were vertically aligned with respect to the gravitational field. Thus, the femur was fixed in space, whereas the tibial segment was only restrained proximally by the contact and ligaments forces. Subsequently, a dummy part together with a bushing element defined between this part and the tibia were used as expedient to drag the tibial segment along with the flexion movement, that is, from 5° extension up to 90° flexion.

Simultaneously, after each iteration, the ligament zero-load lengths were refined until the following conditions were satisfied: during the flexion movement (1) every ligament bundle is stretched, (2) the force exerted by each bundle is below 50 N, and (3) the tibiofemoral contact is maintained^22^.

Furthermore, as necessary, the attachment points of the bundles were also adjusted to agree with the recruitment patterns of the bundles reported in the literature^25^.

### Muscle action

Musculus (M.) quadriceps femoris was also implemented into the model. In particular, M. vastus medialis (V_med_), M. vastus intermedius (V_int_), M. vastus lateralis (V_lat_), and M. rectus femoris (RF) were considered as distinct traction forces (Fig. 1). Origin and insertion muscle attachment points were adapted from data reported in the literature^26^. All muscles originate from the femur (except for the M. rectus femoris which arises from the anterior inferior iliac spine) and insert on the upper edge of the patella through tendons that are modelled as chained ellipsoids. Such tendons representation is important to allow the femoral wrapping mechanism that occurs during the knee flexion. Muscle forces were obtained by means of a proportional–integral–derivative (PID) controller. To take into account the different muscle contributions, the controller output force was multiplied by the specific physiological cross-sectional area (PCSA) (Table 2)^27^ normalized by the mean PCSA (2147 mm^2^). Moreover, the muscle force is conditioned so that only a traction force within a physiological range can be applied. In particular, an upper limit is estimated as the PCSAs of the muscle multiply by maximum isometric muscle stress equal to 1 MPa^28^. A lower force limit is also defined as 1% of the maximum force value^29^. This minimum constant force serves as preload to compensate tendon slack. After the controller tuning process, the proportional, integrative, and derivative K gains resulted equal to 300, 5, and 3, respectively.

**Table 2:** Muscles included in the model with relative PCSAs.

Muscle	PCSA (mm^2^)
M. vastus medialis	2060
M. vastus intermedius	1670
M. vastus lateralis	3510
M. rectus femoris	1350

### Contact definition and pressure distributions

Compliant contacts were also defined between the femoral and patellar components, the femoral component and quadriceps tendons, and the femoral and tibial insert components.

The contact force *F*_*c*_ was computed using an interpenetration formulation derived from Hertz’s contact theory^30^:

3 $$F_{c} = K\delta^{e} + C\left( {\delta , \;\delta_{max} ,\;C_{max} } \right)\dot{\delta }$$

where *K* is the contact stiffness constant in N/mm, *δ* is the penetration depth in mm between the undeformed contacting bodies, *e* is the nonlinear power exponent, $\dot{\delta }$ is the penetration velocity and *C* is a sigmoid damping function that depends on the penetration depth and it is defined by a maximum penetration constant $\delta_{max}$ at which the damping function reaches its maximum value $C_{max}$. Such sigmoid damping functions serve to avoid discontinuities at the initial instant of contact. Hence, on the right side of the equation, the first term represents the elastic force contribute while the second term is the energy dissipated during the contact. In addition, the contribution of the friction force between implant components was taken into account and computed by using a Coulomb’s model considering values of 0.03 and 0.01 as static and dynamic friction coefficients, respectively^31^.

To predict the pressure distribution on the tibial insert, both considered insert geometries were discretized into hexahedral elements with a cross-sectional area (*A*) of 4 mm^2^. Thereby, the elastic foundation theory was used to define the contact stiffness constant between the femoral component and each element of the discretized insert. In particular, the contact stiffness can be expressed as follows:

4 $$K = \frac{(1 - \nu ) E A}{{(1 + \nu )(1 - 2\nu ) h}}$$

with Young’s modulus (*E*) equal to 648 MPa, Poisson’s ratio (ν) of 0.46, and elastic layer thickness (*h*) of 10 mm^32^. Tibiofemoral contact pressures were computed in MATLAB (v. 2021, MathWorks, Natick, MA) as the filtered (zero-phase Butterworth low-pass filter, 2nd order, 5 Hz cut-off frequency) contact forces divided by the cross-sectional area of the insert elements (*A*).

Except for the contact stiffness involved in the tibiofemoral contact, a design of experiment (DOE) was adopted to estimate the contact parameters. Specifically, the objective of the DOE was to minimize the presence of high-frequency oscillations in the monitored contact force of interest, i.e., the tibiofemoral contact force. Table 3 summarizes the final values used for the implementation of the contacts.

**Table 3:** Contact parameters. Value of contact stiffness (*K*), nonlinear power exponent (*e*), maximum damping constant (C_*max*_), maximum penetration constant (δ_*max*_) for the different contact pairs.

Contact pair	*K* (N/mm^e^)	*C*_*max*_ (Ns/mm)	*δ*_*max*_ (mm)	*e*
Femoral component Tibial Insert	1198	375	0.1	1.0
Femoral component Patellar component	30,000	60	0.1	1.5
Femoral component Quadriceps tendon	500	5	0.1	2.0

### Insert designs

The two considered total knee implants are characterized by the same femoral and tibial components, but fixed-bearing polyethylene inserts with different geometries.

Overall, the MP design is characterized by a reduced lateral congruence with respect to the medial one. The purpose of this asymmetry is to replicate the physiological tibiofemoral kinematics, which consists in a tibial internal rotation resulting from the coupled medial pivot and lateral femoral rollback movements during the knee flexion^33^. Conversely, the UC design should guarantee a high congruence between femoral and insert surfaces, over the whole flexion range, showing a specular shape for the medial and lateral compartments.

**Figure 2:**
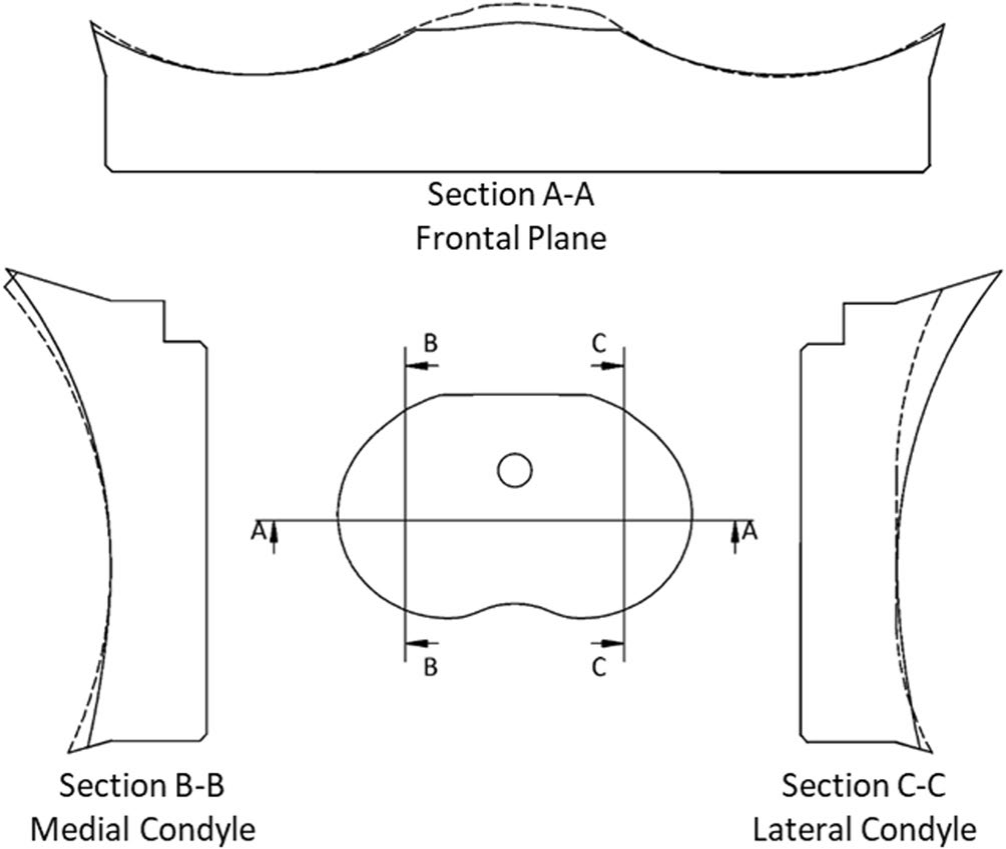
Comparison between the medial-pivot (dashed profiles) and ultra-congruent (solid lines) designs.

However, comparing the sagittal section of each compartment between the designs (Fig. 2), it is possible to see that, on the medial compartment, the MP design is more congruent than the UC, whereas, on the lateral compartment, the MP design shows a significantly lower anterior edge as well as a slightly higher posterior edge with a smaller radius of curvature and, above all, a flat profile at the middle of the compartment. Looking at the insert section on the frontal plane, it can be seen that both compartments of the MP design present a slightly smaller radius of curvature than the UC design.

### Squat simulation

To compare the knee joint dynamics resulting from the different insert designs, a weight-bearing squat simulation was performed, within the gravitational field, by assigning a mass fraction of 29.3 kg to the pelvis. Such mass fraction was derived through the sum of the masses of the body segments (head, neck, upper arm, forearm, hand, trunk, and thigh) computed as a percentage of the whole-body mass^34^ for a male subject of 66.7 kg. In particular, only half of the resulting mass value for trunk, head and neck was taken into account since it was assumed an equal distribution of the load between the two lower limbs.

**Figure 3:**
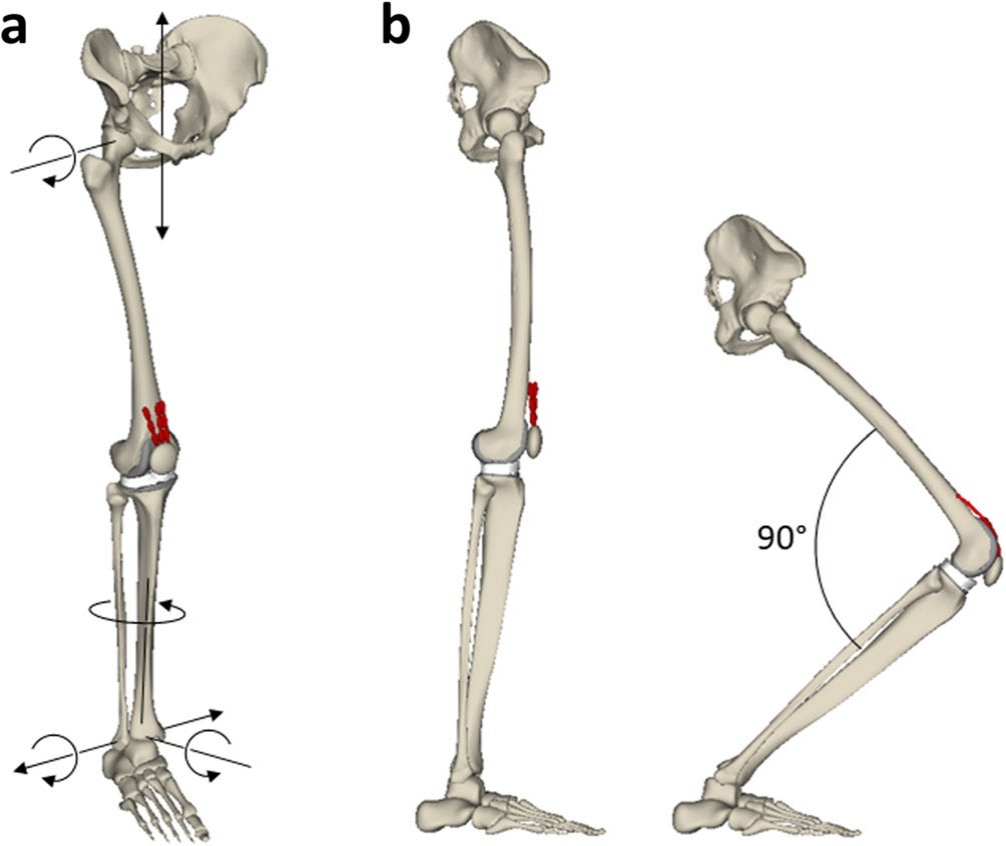
(**a**) Multibody model of the lower right limb with black arrows indicating the DOF assigned to the pelvis, hip, and ankle during the squat simulation; (**b**) lateral view of the model in extension and 90° flexion.

During the squat motion, the pelvis was constrained to translate along the vertical direction, and the flexion movement was allowed at the hip joint (Fig. 3a). All six degrees of freedom (DOF) were permitted for the patellofemoral joint. Moreover, the tibia was constrained to the foot through the ankle joint which was defined to prevent vertical and anteroposterior relative translations^35^. The tibia and fibula were assumed to be one rigid body. Finally, the foot was ground-fixed.

The knee flexion was achieved by defining a specific vertical displacement for the femoral head center, hence, the implemented PID controller continuously adjusted the quadriceps muscle forces to follow the target vertical trajectory, generating a knee flexion from the extended position to 90° flexion (Fig. 3b) in about 2.2 s^36^. This flexion range was chosen since, especially for most patients subjected to TKA, it represents a reasonable range of motion achieved during daily activities.

**Figure 4:**
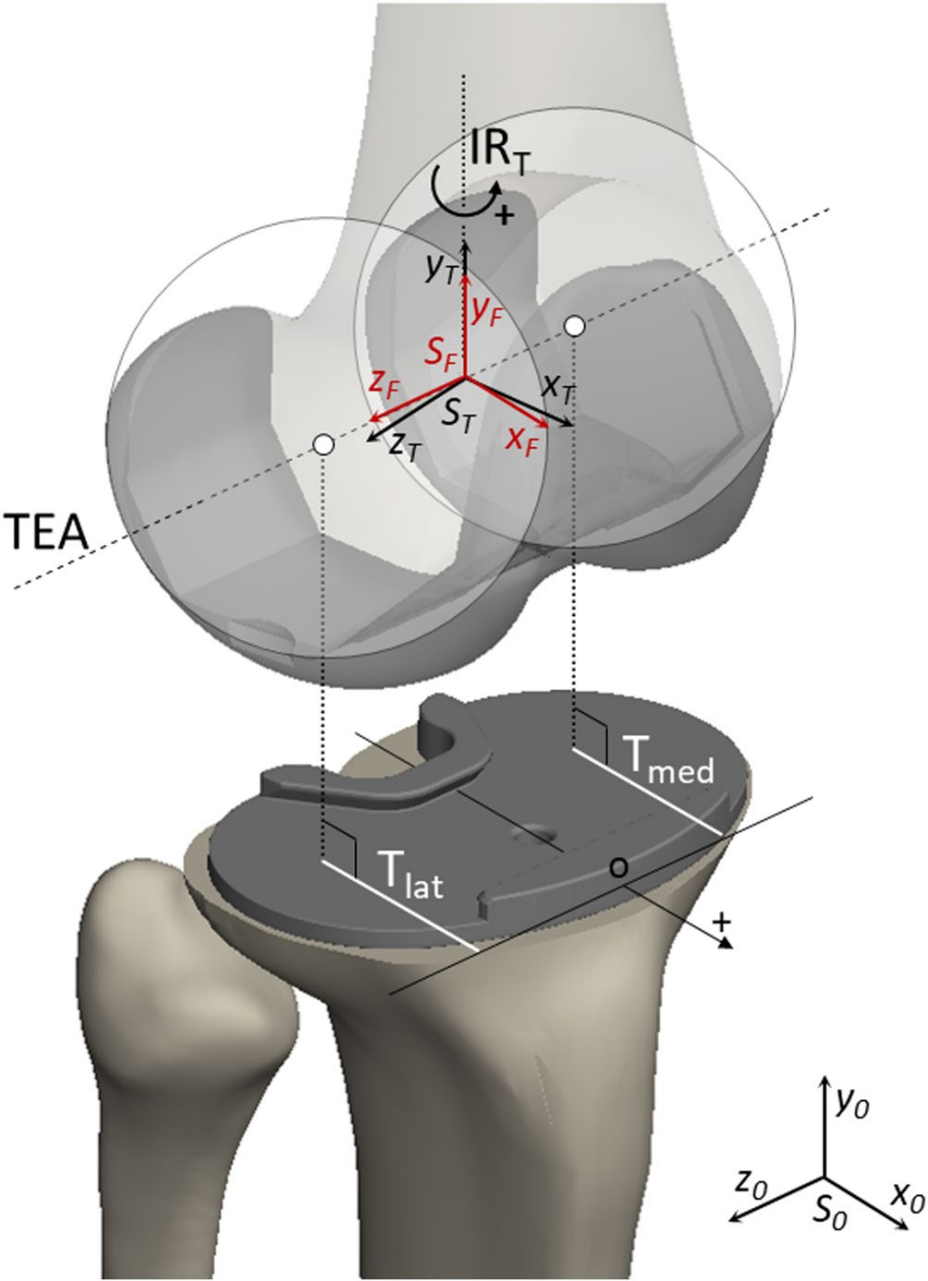
Defined reference systems to measure the tibial internal rotation (IR_T_) and the anteroposterior translation of each condyle. It can be seen the transepicondylar axis (TEA) passing through the centers (white points) of two spheres inscribing the femoral condyles. Also, the two body-fixed reference systems for the femur (S_F_ in red) and the tibia (S_T_ in black) are showed. These are located on the TEA in the middle of the centers of the spheres and used to measure the IR_T_ as the relative rotation of S_T_ with respect to S_F_ along the *y* direction. At the beginning of the simulation, S_F_ and S_T_ are coincident with the y axis along the y_0_ axis of the global reference system (S_0_). The anteroposterior positions of both lateral (T_lat_) and medial (T_med_) condyle are obtained as the projection (white lines) on the tibial tray of the minimal distance between the center of the spheres and the anterior edge of the tibial tray. The condyle position is measured along an axis pointing anteriorly and having origin at the anterior edge of the tibial tray (point O). Therefore, measured positions result negatives.

As regards kinematics measurements, tibial internal rotation was derived from the relative angular displacements between two defined body-fixed reference systems, one for the femur and the other for the tibia (Fig. 4), both with the same orientation and located along the transepicondylar axis (TEA), which is defined as the line passing through the centers of two spheres inscribing the femoral condyles^37^, in the middle of the centers of the spheres.

Moreover, the anteroposterior position of each condyle was measured as the projection on the tibial tray of the minimal distance between the center of the sphere inscribing the condyle and the anterior edge of the tibial component. Based on these defined references, the anteroposterior translation value increases negatively when a femoral condyle moves posteriorly.

## Results

Outcomes from squat simulations, involving the two different insert designs, were compared in terms of tibiofemoral contact force, pressure distribution on the insert surface, anteroposterior translation (femoral rollback) of the lateral and medial femoral condyles, and tibial internal rotation.

**Figure 5:**
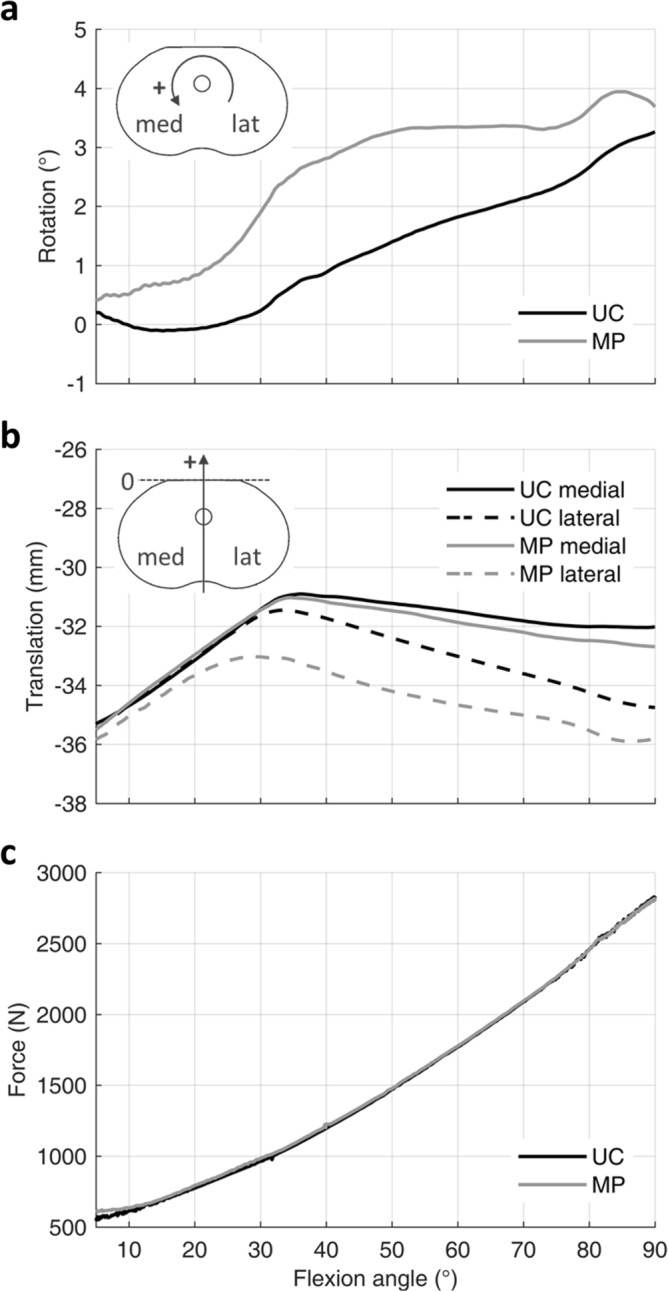
Kinematics and dynamics findings from the squat movement. (**a**) Tibial internal rotation; (**b**) medial and lateral femoral condyles rollback for both MP and UC inserts; (**c**) total tibiofemoral contact force during the squat movement.

Kinematics results showed similar ranges of internal tibial rotation for the MP and UC design, that are, 3.5° and 3.4°, respectively (Fig. 5a). However, it is possible to note a different trend over the whole flexion. Indeed, the UC design generates almost no rotation over the first 30°, maintaining a constant value of about 0°, then the rotation increases linearly up to 3.3°. Conversely, the MP design shows an early increase, reaching 3° of internal rotation at about 45° flexion, successively, the rotation stays between 3° and 4°.

Overall, the femoral rollback measurements show an anterior translation, over the first 35°, followed by a posterior translation (Fig. 5b). In detail, both designs generate a medial anterior translation of about 4.4 mm, whereas the lateral anterior translation results equal to 3.9 mm for the UC and only 2.3 mm for the MP. Starting from 35° flexion, in both designs, the lateral femoral condyle performs a rollback that recovers the previous anterior translation. On the other side, the medial posterior translation is limited to 1.1 mm and 1.8 mm for the UC and MP, respectively.

Concerning the tibiofemoral contact forces (Fig. 5c), values equal to 561 N and 609 N were obtained in extension for the UC and MP, respectively. For both designs the contact force increases over the whole knee flexion, leading to a maximum common value of about 2824 N at 90° flexion, which corresponds to 4.3 times the total BW.

Although no remarkable differences between UC and MP design were observed in total tibiofemoral contact forces, the relative contact pressure distributions on the insert surface revealed considerable distinctions.

**Figure 6:**
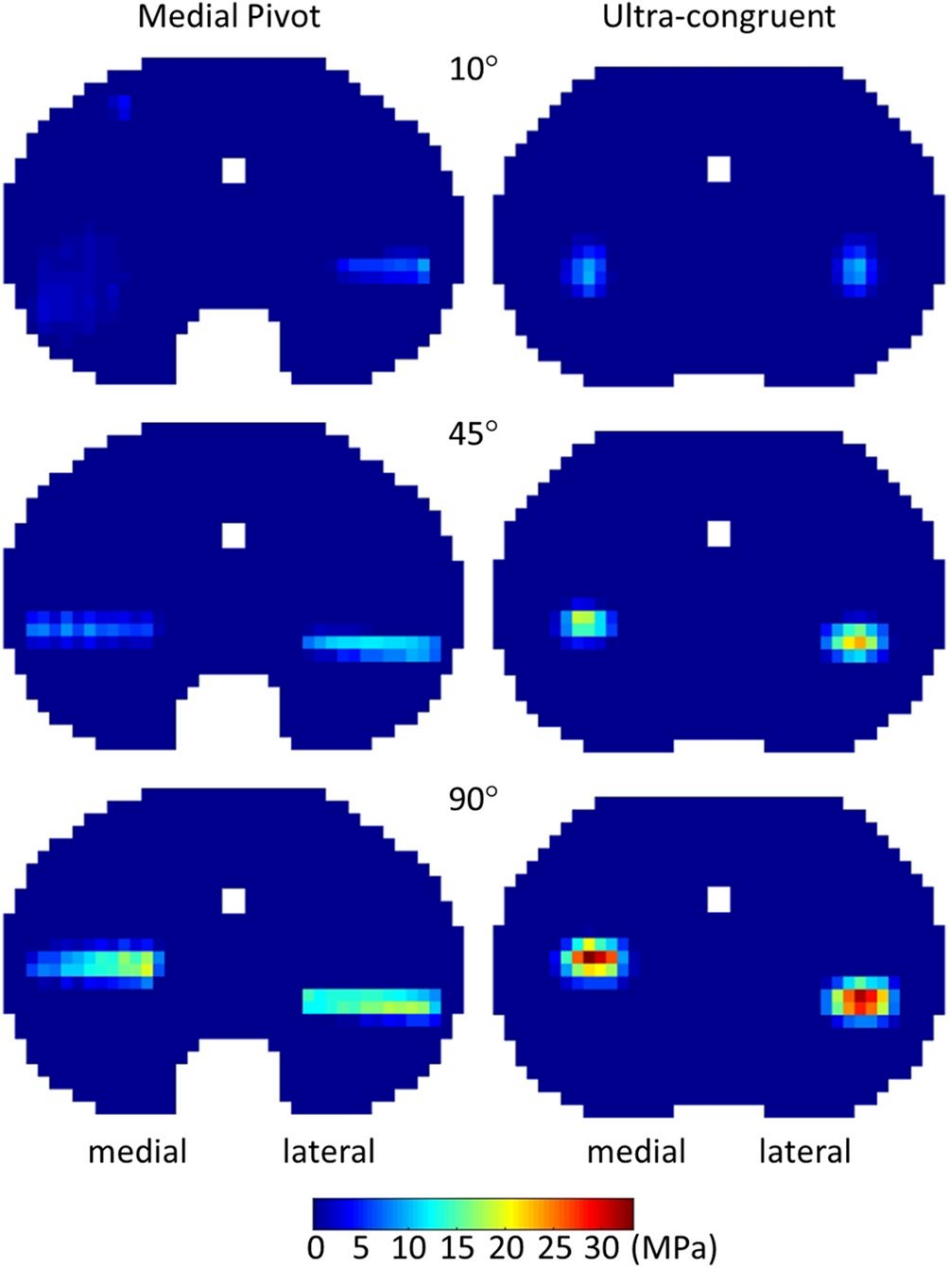
Pressure distribution on the insert surface at three successive flexion angles (10°, 45° and 90°) during the squat movement.

Overall, contact pressures increase together with the flexion angle, reaching the highest values at 90° flexion (Fig. 6). Nevertheless, the UC design causes more concentrated contact forces than the MP design, on both medial and lateral insert compartments. Specifically, the UC design presents narrower and more rounded contact footprints, whereas the MP design is characterized by wider contact footprints. In particular, the MP design presents a well-distributed contact on the medial compartment for 10° flexion. Peak pressures resulted equal to 20 MPa and 33 MPa for MP and UC design, respectively. Such peak values were found on the medial compartment in both designs. Furthermore, it is possible to note that the lateral contact moves posteriorly, in contrast with the medial one, which moves anteriorly over the flexion.

## Discussion

The presented computational study allowed for a realistic biomechanical investigation of the behaviour of a total knee arthroplasty with two different shapes of the insert component, namely, the MP and UC designs. Kinematics results revealed that the alternative designs generate a different trend of the tibial internal rotation. Especially, during the first 30° of flexion, the MP design showed a more physiological behaviour allowing an immediate and gradual tibial internal rotation, while the UC design seems to hinder the rotation in the same flexion range. Compared to the physiological knee, findings showed a reduced internal tibial rotation due to the TKA, substantially confirming what was reported in the literature. Indeed, considering a 90° squat task, Dennis et al.^38^ observed an average maximum internal tibial rotation of 17.5° in healthy knees, and 2.7° in patients having a fixed-bearing posterior-cruciate-retaining TKA with the posterior cruciate ligament sacrificed.

Regarding the femoral rollback, during early knee flexion, it can be noted the so-called “paradoxical motion”, that is, the anterior translation of the femoral condyles^39^. Such anterior femoral sliding, although being not physiological, was observed in most of the total knee designs and should be carefully considered since it may lead to patient dissatisfaction^6,40,41^. Moreover, a similar amplitude of lateral rollback between designs was observed. The reason behind this result could be a low difference between the designs in terms of the posterior profile, on the sagittal plane, of the lateral compartment. The distinctive kinematics owing to the designs might have been also mitigated by the equally applied boundary conditions, such as the lack of cruciate ligaments.

Concerning the tibiofemoral contact forces, no considerable differences arose between the different designs. However, during extension, the MP design generates a slightly higher force (+ 8.5%) than the UC. This difference may be ascribed to the higher sagittal conformity of the medial compartment of the MP design, which leads to a greater restraining effect^3^. The maximum contact force of 4.3 BW is within the range of 2.3–7.3 BW reported in the literature^42^.

The implementation of the discretization process applied to the insert geometry permitted to obtain the contact pressure distributions on the surface of the inserts, directly within the multibody simulation framework. In this regard, with respect to the MP design, the UC design causes force concentrations which could trigger or speed up wearing phenomena. This is owing to the greater radius of curvature of the medial and lateral compartments that the UC design presents on the frontal plane. Indeed, even if minimal, such difference produces lower conformity on the frontal plane between the femoral condyles and insert compartments, explaining the more rounded shape of the contact footprints observed for the UC design. Conversely, the higher conformity of the MP design on the frontal plane, justifies the wider contact footprints extended along the mediolateral axis. Looking at the maximum pressure values, 20 MPa and 33 MPa for MP and UC design, respectively, these are consistent with findings of other numerical studies. For instance, Bei and Fregly^43^ reported a peak pressure of 28 MPa, during a gait cycle, under a load of 3 BW. In their experiments, Fregly and co-workers^44^ measured pressure peaks between 25 and 35 MPa, under a loading condition of 3000 N at 90° flexion. Furthermore, Stylianou predicted a maximum value of about 26 MPa at 2500 N of tibiofemoral compressive force during a squat motion^31^. Also, in the numerical study of Shu and colleagues, a maximum contact pressure of approximately 35 MPa was observed for a low conformity design during squatting^45^. However, it has to be stated that direct comparisons with other studies should be carefully interpreted since findings are highly design-dependent, especially those related to the contact pressure.

Overall, this study presents some limitations that need to be pointed out. First, ligaments' locations were derived by anatomical references. A better approach would consist in deriving the ligaments' attachment points directly from magnetic resonance imaging (MRI) images. Second, although in line with methods reported by other studies, only the quadriceps muscle group was here implemented. In future developments, additional muscles will be integrated into the model (e.g., the hamstrings). Furthermore, the performed weight-bearing squat simulation, with its specifically constraints set, cannot exactly mimic a squat movement as performed in vivo^46^. Rather, this latter could be achieved by integrating motion capture data into the model. On the other hand, the implemented squat simulation allows for realistic knee loading conditions while resulting particularly suitable for being validated in vitro by means of experimental setups, i.e., test rigs^47–49^.

Finally, it should be stated that findings were not directly validated against ad hoc experiments; however, the conducted comparison with reported data was performed showing good agreements with the literature. Moreover, thanks to the discretization of the insert geometry, the contact locations, as well as the pressure maps on the insert surface, were efficiently obtained within the multibody framework, therefore, without the need of further finite element analyses (FEA) which, as known, implies intensive computational efforts.

## Conclusions

In conclusion, the here presented findings can provide indications for total knee implants development and optimization, highlighting the essential role of both sagittal and frontal tibiofemoral conformity in order to avoid pressure peaks, which might lead to the insert deterioration due to yielding phenomena. In particular, the considered MP design, with respect to the UC, showed a more physiological-like tibial rotation in the early knee flexion and lower tibiofemoral contact pressures, ultimately suggesting a reduced wear rate and, therefore, an improved implant longevity.

## Data Availability

All data generated and/or analysed during this study are available from the corresponding author on reasonable request except for the insert geometries, which were used under license from Gruppo Bioimpianti (Peschiera Borromeo, Milan, Italy) for the current study, and so are not publicly available.
